# Inhibition of poly(I:C)–induced matrix metalloproteinase expression in human corneal fibroblasts by triptolide

**Published:** 2011-02-18

**Authors:** Kazuhiro Kimura, Norimasa Nomi, Zhou Hong Yan, Tomoko Orita, Teruo Nishida

**Affiliations:** Department of Ophthalmology, Yamaguchi University Graduate School of Medicine, Ube City, Yamaguchi, Japan

## Abstract

**Purpose:**

Triptolide is a major component of the herb *Tripterygium wilfordii* Hook f, extracts of which are used in traditional Chinese medicine, and it has been found to possess immunosuppressive and anti-inflammatory properties. Viral infection of the cornea can lead to corneal ulceration and perforation as a result of collagen degradation in the corneal stroma. We have now examined the effect of triptolide on the expression of matrix metalloproteinases (MMPs) induced by polyinosinic-polycytidylic acid [poly(I:C)], a synthetic analog of viral double-stranded RNA, in cultured human corneal fibroblasts.

**Methods:**

Human corneal fibroblasts were cultured in the absence or presence of poly(I:C) or triptolide. Secretion of MMPs as well as the phosphorylation of mitogen-activated protein kinases (MAPKs) and the NF-κB–inhibitory protein, IκB-α, were examined by immunoblot analysis. The abundance of *MMP* mRNAs was determined by reverse transcription and real-time polymerase chain reaction analysis.

**Results:**

Poly(I:C) induced the secretion of MMP-1 and MMP-3 from corneal fibroblasts in a concentration-dependent manner as well as increased the intracellular abundance of *MMP-1* and *MMP-3* mRNAs. Triptolide inhibited these effects of poly(I:C) on MMP expression in a concentration-dependent manner. The poly(I:C)-induced secretion of MMP-1 and MMP-3 was also attenuated by synthetic inhibitors of MAPK and NF-κB signaling pathways. Triptolide inhibited the poly(I:C)-induced phosphorylation of IκB-α but did not affect that of the MAPKs, Extracellular Signal-Regulated Kinase (ERK), p38MAPK, and c-Jun N-Terminal Kinase (JNK).

**Conclusions:**

Triptolide inhibited the poly(I:C)-induced production of MMP-1 and MMP-3 by human corneal fibroblasts. Triptolide therefore warrants further investigation as a potential treatment for corneal ulceration associated with viral infection.

## Introduction

Viral infection of the cornea induces local inflammation that can result in damage to the corneal stroma, including corneal ulceration and perforation [1,2]. Collagen degradation in the corneal stroma contributes to corneal ulceration associated with viral infection. Matrix metalloproteinases (MMPs) are released from cells in the form of proenzymes (proMMPs) and are activated by proteolytic processing in response to various stimuli [3,4]. These proteinases play a key role in the degradation of extracellular matrix proteins and are released by both resident and infiltrated cells in association with inflammation [5-10]. Corneal fibroblasts (activated keratocytes) produce MMPs in response to certain stimuli [11,12], with collagenase (MMP-1), stromelysin (MMP-3), and gelatinase (MMP-2) enzymes having been shown to be secreted by these cells in response to stimuli associated with corneal ulceration [13-17].

Triptolide is a major component of extracts of the plant *Tripterygium wilfordii* Hook f, which have been used in traditional Chinese medicine. Triptolide has been found to have immunosuppressive and anti-inflammatory properties [18,19]. It has thus been shown to inhibit the production of various cytokines and chemokines by immune and other cell types in association with inflammation [20,21]. We have previously shown that triptolide inhibits the expression of cytokines, chemokines, and adhesion molecules induced by the bacterial component lipopolysaccharide in rabbit corneal fibroblasts [6].

We have also shown that polyinosinic-polycytidylic acid [poly(I:C)], a synthetic analog of viral double-stranded RNA, induces the production of cytokines, chemokines, and adhesion molecules in human corneal fibroblasts [7]. In addition, we previously investigated the effect of poly(I:C) on MMP expression in human corneal fibroblasts to provide insight into the role of these enzymes in corneal ulceration associated with viral infection. We found that poly(I:C) increased the expression of MMP-1 and MMP-3 in these cells [11]. Although patients with viral corneal ulceration are treated with antiviral agents, drugs that prevent the progression of corneal stromal melting or perforation remain to be discovered. We have therefore now examined the effect of triptolide on MMP expression in human corneal fibroblasts exposed to poly(I:C) to investigate whether this agent might be a potential treatment for viral corneal ulcer.

## Methods

### Materials

Eagle’s minimum essential medium (MEM), fetal bovine serum, and Trizol reagent were obtained from Invitrogen-Gibco (Carlsbad, CA), and 24-well culture plates and 60-mm culture dishes were from Corning-Costar (Corning, NY). Poly(I:C) was obtained from Invivogen (San Diego, CA), and triptolide was from Allexis Biochemicals (Carlsbad, CA). A reverse transcription (RT) system was from Promega (Madison, WI). PD98059, SB203580, c-Jun NH2-terminal kinase (JNK) inhibitor II, and I-kappa-B Kinase Beta (IKK-2) inhibitor were obtained from Calbiochem (La Jolla, CA). A protease inhibitor cocktail was from Sigma-Aldrich (St. Louis, MO). Mouse monoclonal antibodies to MMP-1 or to MMP-3 were obtained from Daiichi Fine Chemicals (Toyama, Japan). Rabbit polyclonal antibodies to total or phosphorylated forms of extracellular signal–regulated kinase (ERK), p38 mitogen-activated protein kinase (MAPK), JNK, or I kappa B-alpha (IκB-α) were obtained from Cell Signaling (Beverly, MA), and rabbit polyclonal antibodies to the p65 subunit of Nuclear Factor-kappa B (NF-κB) were from Santa Cruz Biotechnology (Santa Cruz, CA). TOTO-3 and AlexaFluor 488–labeled goat antibodies to rabbit immunoglobulin G were from Invitrogen. Horseradish peroxidase–conjugated secondary antibodies, nitrocellulose membranes, and an enhanced chemiluminescence (ECL) kit were obtained from GE Healthcare (Uppsala, Sweden).

### Isolation and culture of human corneal fibroblasts

Human corneas obtained for corneal transplantation surgery from NorthWest Lions Eye Bank (Seattle, WA) were used in accordance with the tenets of the Declaration of Helsinki. Corneal fibroblasts were prepared from the stromal tissue remaining after transplantation and were cultured as described previously [5]. In brief, the endothelial layer of the cornea was removed mechanically before treatment of the tissue with dispase (2 mg/ml in MEM) for 1 h at 37 °C. The epithelial sheet was then removed from the tissue before its further exposure to collagenase (2 mg/ml in MEM) at 37 °C to obtain a single-cell suspension. The isolated cells were maintained under a humidified atmosphere of 5% CO2 at 37 °C in MEM supplemented with 10% fetal bovine serum. The cells were used for experiments after four to six passages.

### Immunoblot analysis

For analysis of MMP secretion, cells were incubated in 24-well plates with various concentrations of poly(I:C) for 24 h, after which the culture supernatants were collected and subjected to SDS–PAGE on a 10% gel. For analysis of the activation of signaling molecules, cell lysates were prepared after exposure of the cells to poly(I:C) for 1 h and were also subjected to SDS–PAGE. After electrophoresis, the separated proteins were transferred to a nitrocellulose membrane, which was then exposed to blocking solution (20 mM Tris-HCl [pH 7.4], 5% dried skim milk, 0.1% Tween-20) before incubation for 16 h at 4 °C with primary antibodies at a 1:1,000 dilution in blocking solution. The membrane was washed with a solution containing 20 mM Tris-HCl (pH 7.4) and 0.1% Tween-20, incubated for 1 h at room temperature with horseradish peroxidase–conjugated secondary antibodies at a 1:1,000 dilution in the same solution, washed again, incubated with ECL reagents for 5 min, and then exposed to film.

### RT and real-time PCR analysis

Total RNA was isolated from cells in 60-mm culture dishes with the use of the Trizol reagent and was subjected to RT. The resulting cDNA was subjected to real-time polymerase chain reaction (PCR) analysis with the use of a LightCycler instrument (Roche Molecular Biochemicals, Indianapolis, IN) and with primers specific for *MMP-1*, *MMP-3*, or glyceraldehyde-3-phosphate dehydrogenase (*GAPDH*), as previously described [22,23] and shown in Table 1. Transcripts of the constitutively expressed gene for *GAPDH* served to normalize the amounts of *MMP-1* and *MMP-3* mRNAs in each sample. Real-time PCR data were analyzed with LightCycler ver. 3.1 software (Roche Molecular Biochemicals).

**Table 1:** PCR primer sets.

**Gene**	**Primer sequence (5′-3′)**
*MMP-1*	CGACTCTAGAAACACAAGAGCAAGA(sense)
	AAGGTTAGCTTACTGTCACACGCTT (antisense)
*MMP-3*	GGCACAATATGGGCACTTTA (sense)
	CCGGCAAGATACAGATTCAC (antisense)
*G3PDH*	GCCAAAAGGGTCATCATCTC (sense)
	ACCACCTGGTGCTCAGTGTA (antisense)

### Immunofluorescence staining

Immunostaining for the p65 subunit of NF-κB was performed as described previously [24]. Cells in 24-well plates were washed twice with phosphate-buffered saline (PBS), fixed with 4% paraformaldehyde in PBS, and washed an additional three times with PBS before permeabilization with 100% methanol for 6 min at –20 °C. The cells were exposed to PBS containing 3% BSA for 30 min before incubation for 1 h at room temperature with antibodies to the p65 subunit of NF-κB. They were then washed, incubated for 30 min at room temperature with AlexaFluor 488–conjugated goat secondary antibodies and TOTO-3 (1:1,000 dilution), washed again, and finally examined with a laser confocal microscope (LSM5; Carl Zeiss, Hallbergmoos, Germany).

### Statistical analysis

Quantitative data are presented as means±SEM. Differences were analyzed by Dunnett’s multiple comparison test. A p value of <0.05 was considered statistically significant.

## Results

**Figure 1:**
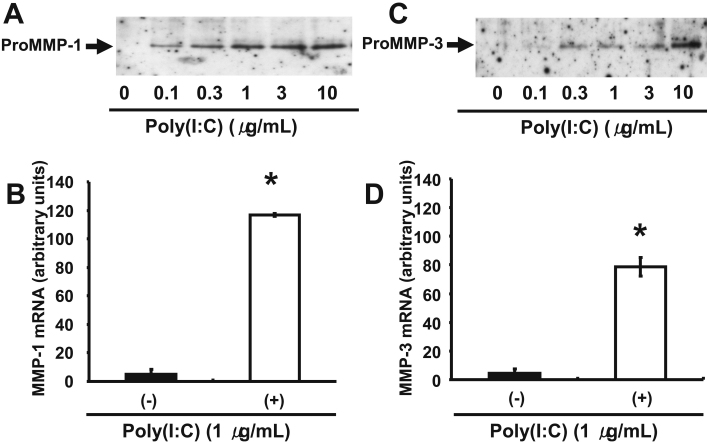
Effect of poly(I:C) on the expression of MMP-1and MMP-3 in human corneal fibroblasts. **A**, **C**: Cells were incubated for 24 h in the presence of the indicated concentrations of poly(I:C), after which the culture supernatants were collected and subjected to immunoblot analysis with antibodies to MMP-1 (**A**) and to MMP-3 (**C**). The positions of the bands corresponding to proMMP-1 and proMMP-3 are indicated. Data are representative of three independent experiments. **B**, **D**: Cells were incubated for 24 h in the absence or presence of poly(I:C) at 1 µg/ml, after which total RNA was isolated and subjected to RT and real-time PCR analysis of *MMP-1* (**B**) and *MMP-3* (**D**) mRNAs. Data were normalized by the abundance of *GAPDH* mRNA and are presented in arbitrary units; they are means±SEM from three separate experiments. *p<0.05 (Dunnett’s test) versus the corresponding value for cells cultured without poly(I:C).

**Figure 2:**
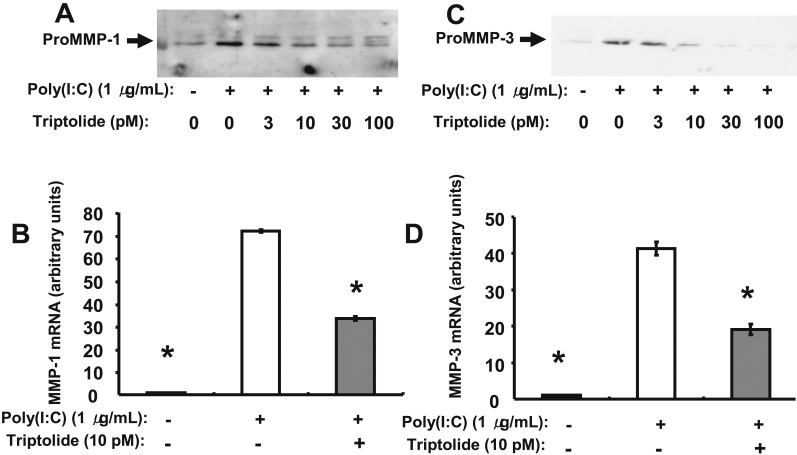
Effect of triptolide on the poly(I:C)-induced expression of MMP-1 and MMP-3 in human corneal fibroblasts. **A**, **C**: Cells were incubated for 1 h in the presence of the indicated concentrations of triptolide and then for 24 h in the additional absence or presence of poly(I:C) at 1 µg/ml. The culture supernatants were then collected and subjected to immunoblot analysis with antibodies to MMP-1 (**A**) and to MMP-3 (**C**). Data are representative of three independent experiments. **B**, **D**: Cells were incubated for 1 h in the absence or presence of triptolide (10 pM) and then for 24 h in the additional absence or presence of poly(I:C) at 1 µg/ml. Total RNA was then isolated from the cells and subjected to RT and real-time PCR analysis of *MMP-1* (**B**) and *MMP-3* (**D**) mRNAs. Data were normalized by the abundance of *GAPDH* mRNA and are presented in arbitrary units; they are means±SEM from three separate experiments. *p<0.05 (Dunnett’s test) versus the corresponding value for cells cultured with poly(I:C) but without triptolide.

We first examined the effect of poly(I:C) at various concentrations (0 to 10 μg/ml) on the expression of MMP-1 and MMP-3 in human corneal fibroblasts. Immunoblot analysis showed that incubation of the cells with poly(I:C) for 24 h induced the secretion of proMMP-1 and proMMP-3 in a concentration-dependent manner (Figure 1A,C), consistent with our previous observations [11]. Furthermore, quantitative RT–PCR analysis revealed that the amounts of *MMP-1* and *MMP-3* mRNAs in corneal fibroblasts were increased by incubation of the cells with poly(I:C) at 1 µg/ml for 24 h (Figure 1B,D). We next examined the effect of triptolide on the upregulation of *MMP-1* and *MMP-3* expression in human corneal fibroblasts by poly(I:C). Immunoblot analysis showed that triptolide inhibited the poly(I:C)-induced increase in proMMP-1 and proMMP-3 secretion from these cells in a concentration-dependent manner, with the maximal effect apparent at a triptolide concentration of ~30 pM (Figure 2A,C). In addition, quantitative RT–PCR analysis revealed that the increase in the amounts of *MMP-1* and *MMP-3* mRNAs induced by poly(I:C) in human corneal fibroblasts was inhibited by triptolide at 10 pM (Figure 2B,D).

**Figure 3:**
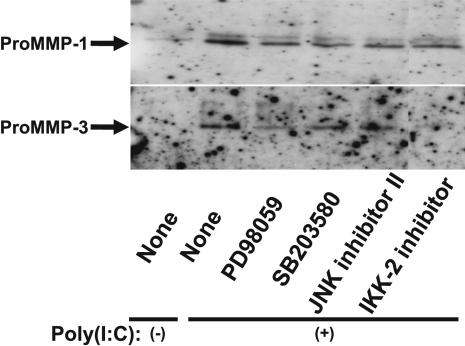
Effects of inhibitors of MAPK or NF-κB signaling on the poly(I:C)-induced expression of MMP-1 and MMP-3 in human corneal fibroblasts. Cells were incubated first for 1 h in the absence or presence of PD98059, SB203580, or JNK inhibitor II (each at 10 µM) or of IKK-2 inhibitor (1 µM) and then for 24 h in the additional absence or presence of poly(I:C) at 1 µg/ml. The culture supernatants were then collected and subjected to immunoblot analysis with antibodies to MMP-1 and to MMP-3. Data are representative of three independent experiments.

We previously showed that poly(I:C) activates the MAPKs ERK, p38, and JNK as well as the NF-κB signaling pathway in human corneal fibroblasts [7]. We therefore examined the possible role of these signaling molecules in the upregulation of *MMP-1* and *MMP-3* expression in human corneal fibroblasts exposed to poly(I:C). Cells were incubated first with the ERK signaling inhibitor PD98059, the p38 inhibitor SB203580, or JNK inhibitor II (each at 10 μM) or with IκB kinase–2 (IKK-2) inhibitor (1 μM) for 1 h and then in the additional presence of poly(I:C) at 1 µg/ml for 24 h. Immunoblot analysis showed that the poly(I:C)-induced release of proMMP-1 and proMMP-3 from corneal fibroblasts was inhibited by each of the three MAPK inhibitors as well as by IKK-2 inhibitor (Figure 3), the latter of which blocks signaling by the NF-κB pathway.

**Figure 4:**
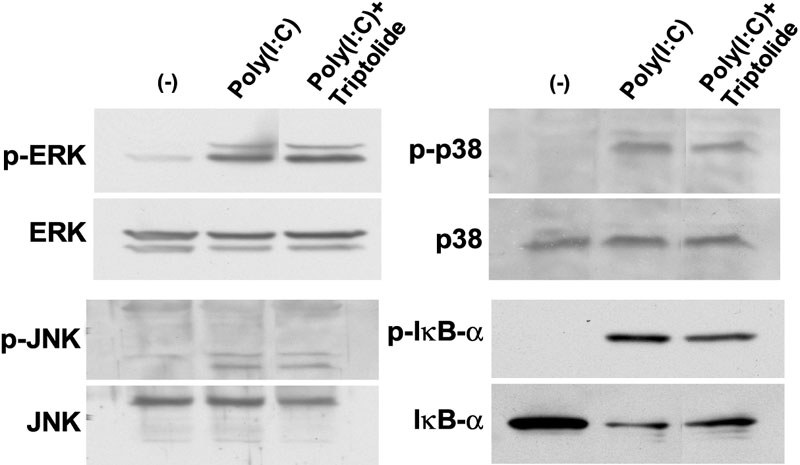
Effect of triptolide on the poly(I:C)-induced activation of MAPK and NF-κB signaling pathways in human corneal fibroblasts. Cells were incubated first for 1 h in the absence or presence of triptolide (10 pM) and then for 1 h in the additional absence or presence of poly(I:C) at 1 µg/ml. Cell lysates were then prepared and subjected to immunoblot analysis with antibodies to total or phosphorylated (p-) forms of ERK, p38, JNK, or IκB-α. Data are representative of three independent experiments.

**Figure 5:**
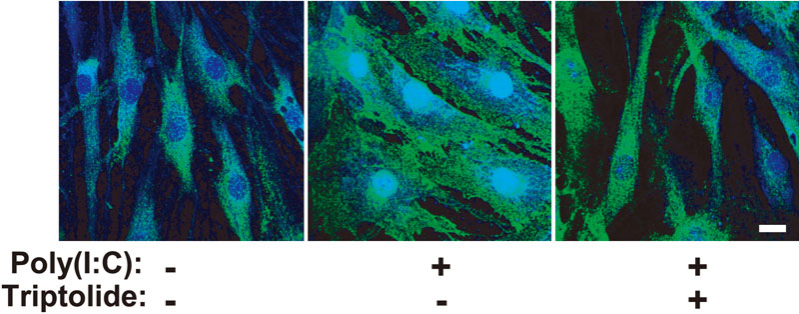
Effect of triptolide on the poly(I:C)-induced activation of NF-κB in human corneal fibroblasts. Cells were incubated first for 1 h in the absence or presence of triptolide (3 nM) and then for 1 h in the additional absence or presence of poly(I:C) at 1 μg/ml. The cells were then subjected to immunofluorescence analysis with antibodies to the p65 subunit of NF-κB (green fluorescence), and nuclei were stained with TOTO-3 (blue fluorescence). Scale bar, 20 µm. Data are representative of three independent experiments.

We next examined the possible effect of triptolide on poly(I:C)-induced signaling by MAPK and NF-κB pathways. Immunoblot analysis revealed that triptolide inhibited the phosphorylation and degradation of the endogenous NF-κB inhibitor IκB-α induced by poly(I:C), whereas it had no effect on the poly(I:C)-induced phosphorylation (activation) of the MAPKs ERK, p38, or JNK (Figure 4). Finally, immunofluorescence analysis showed that incubation of the cells with poly(I:C) at 1 µg/ml for 1 h induced translocation of the p65 subunit of NF-κB from the cytosol to the nucleus and that this effect was inhibited by triptolide at 3 nM (Figure 5).

## Discussion

We have shown that the expression of *MMP-1* and *MMP-3* at the mRNA and protein levels is increased in human corneal fibroblasts exposed to poly(I:C), a synthetic analog of viral double-stranded RNA, consistent with our previous observations [11]. Triptolide inhibited the poly(I:C)-induced expression of *MMP-1* and *MMP-3* at both the mRNA and protein levels in these cells. The expression of *MMP-1* and *MMP-3* induced by poly(I:C) was also attenuated by MAPK inhibitors and IKK-2 inhibitor. Whereas poly(I:C) induced the phosphorylation of MAPKs and IκB-α in corneal fibroblasts, only that of IκB-α was inhibited by triptolide. MMPs play an important role in the degradation of extracellular matrices in various tissues and contribute to the pathogenesis of a variety of diseases [25-27]. The expression of MMPs with collagenolytic and gelatinolytic activities has been detected in association with bacterial corneal ulcer [28,29]. MMP-1 and MMP-3 were also detected in tear fluid and corneal tissue affected by bacterial keratitis [30]. We have previously shown that poly(I:C) induces the expression of *MMP-1* and *MMP-3* in human corneal fibroblasts [11]. The expression of MMPs is also induced in tissue affected by viral keratitis [31,32]. MMPs also contribute to tissue infiltration by polymorphonuclear leukocytes [33]. The adherence of neutrophils to extracellular matrix inhibits their activation [34]. These observations thus suggest that the activation of MMPs in viral keratitis may induce the remodeling of extracellular matrix and promote the infiltration of polymorphonuclear leukocytes into the cornea, eventually leading to the development of corneal ulcer. Additional experiments with physiologic animal models or viral agents such as herpes simplex virus should help to clarify the mechanism of viral corneal ulceration and the effect of triptolide on this process.

Corneal fibroblasts upregulate the expression of cytokines, chemokines, and adhesion molecules in response to various stimuli including poly(I:C) [7], suggesting that these cells may play an important role in inflammation of the corneal stroma induced by viral infection. We previously showed that the poly(I:C)-induced expression of these molecules in human corneal fibroblasts is mediated by MAPK and NF-κB signaling pathways [7]. We also previously showed that the poly(I:C)-induced expression of *MMP-1* and *MMP-3* in human corneal fibroblasts is mediated at least in part by the NF-κB signaling pathway [11]. In the present study, we found that the poly(I:C)-induced upregulation of *MMP-1* and *MMP-3* expression was also attenuated by inhibitors of ERK, p38, and JNK signaling. However, whereas triptolide inhibited activation of the NF-κB signaling pathway induced by poly(I:C) in human corneal fibroblasts, it did not affect the activation of MAPK signaling pathways. The poly(I:C)-induced expression of *MMP-1* and *MMP-3* in corneal fibroblasts is thus dependent on both MAPK and NF-κB signaling pathways, but triptolide appears to attenuate the upregulation of *MMP-1* and *MMP-3* specifically through inhibition of NF-κB signaling. The cytokines tumor necrosis factor–α and interleukin (IL)–1β were previously shown to induce *MMP* expression in an NF-κB–dependent manner in synovial fibroblasts and chondrocytes, respectively [35,36]. Triptolide was also previously shown to inhibit the lipopolysaccharide-induced expression of IL-12 and IL-23 in macrophages [37] as well as the expression of IL-18 in synovial fibroblasts [38]. We previously showed that the poly(I:C)-induced expression of *MMP-1* and *MMP-3* in corneal fibroblasts is mediated in part by secreted IL-1β [11]. The effects of triptolide on the expression of cytokines, chemokines, and adhesion molecules in corneal fibroblasts induced by poly(I:C) remain to be determined.

Corneal ulceration associated with viral keratitis can result in corneal scarring and perforation. Viral infection of the cornea is typically treated with antiviral eyedrops. However, such treatment does not always result in the healing or prevent the progression of corneal ulcer. Corneal ulceration results from the destruction of stromal collagen by MMPs and other proteases, and we have now shown that triptolide inhibits the expression of MMP*-1* and *MMP-3* induced by poly(I:C) in human corneal fibroblasts. Although further studies are required to determine the effect of triptolide on viral corneal ulceration, our results suggest that triptolide might prove effective as a new drug for the treatment of this condition.
